# Force-Induced Strengthening of the Interaction between *Staphylococcus aureus* Clumping Factor B and Loricrin

**DOI:** 10.1128/mBio.01748-17

**Published:** 2017-12-05

**Authors:** Pauline Vitry, Claire Valotteau, Cécile Feuillie, Simon Bernard, David Alsteens, Joan A. Geoghegan, Yves F. Dufrêne

**Affiliations:** a Institute of Life Sciences, Université Catholique de Louvain, Louvain-la-Neuve, Belgium; b Department of Microbiology, Moyne Institute of Preventive Medicine, School of Genetics and Microbiology, Trinity College Dublin, Dublin, Ireland; c Walloon Excellence in Life Sciences and Biotechnology (WELBIO), Wavre, Belgium; University of Washington

**Keywords:** atomic force microscopy, cell adhesion, physical stress, skin, *Staphylococcus aureus*

## Abstract

Bacterial pathogens that colonize host surfaces are subjected to physical stresses such as fluid flow and cell surface contacts. How bacteria respond to such mechanical cues is an important yet poorly understood issue. *Staphylococcus aureus* uses a repertoire of surface proteins to resist shear stress during the colonization of host tissues, but whether their adhesive functions can be modulated by physical forces is not known. Here, we show that the interaction of *S. aureus* clumping factor B (ClfB) with the squamous epithelial cell envelope protein loricrin is enhanced by mechanical force. We find that ClfB mediates *S. aureus* adhesion to loricrin through weak and strong molecular interactions both in a laboratory strain and in a clinical isolate. Strong forces (~1,500 pN), among the strongest measured for a receptor-ligand bond, are consistent with a high-affinity “dock, lock, and latch” binding mechanism involving dynamic conformational changes in the adhesin. Notably, we demonstrate that the strength of the ClfB-loricrin bond increases as mechanical force is applied. These findings favor a two-state model whereby bacterial adhesion to loricrin is enhanced through force-induced conformational changes in the ClfB molecule, from a weakly binding folded state to a strongly binding extended state. This force-sensitive mechanism may provide *S. aureus* with a means to finely tune its adhesive properties during the colonization of host surfaces, helping cells to attach firmly under high shear stress and to detach and spread under low shear stress.

## IMPORTANCE

*Staphylococcus aureus* colonizes the human skin and the nose and can cause various disorders, including superficial skin lesions and invasive infections. During nasal colonization, the *S. aureus* surface protein clumping factor B (ClfB) binds to the squamous epithelial cell envelope protein loricrin, but the molecular interactions involved are poorly understood. Here, we unravel the molecular mechanism guiding the ClfB-loricrin interaction. We show that the ClfB-loricrin bond is remarkably strong, consistent with a high-affinity “dock, lock, and latch” binding mechanism. We discover that the ClfB-loricrin interaction is enhanced under tensile loading, thus providing evidence that the function of an *S. aureus* surface protein can be activated by physical stress.

## INTRODUCTION

*Staphylococcus aureus* is a commensal Gram-positive bacterium that colonizes the nares and skin of humans (1). Nasal carriage of *S. aureus* is of great medical significance, as it is a major risk for infection (2–4). Skin colonization may be associated with various disorders, including skin infections, atopic dermatitis (AD), endocarditis, and septicemia (5–7). *S. aureus* attaches to corneocytes or squamous epithelial cells via cell wall-anchored (CWA) proteins, including iron-regulated surface determinant A (IsdA) (8) and clumping factor B (ClfB) (9–11). Currently, the molecular details of these interactions are poorly understood. Also, whether the adhesive properties of CWA proteins change in response to mechanical force is not known. Solving this problem would help us understand how bacteria colonize mucosal surfaces while being subjected to various physical stresses, including fluid flow and cell surface interactions.

**FIG 1:**
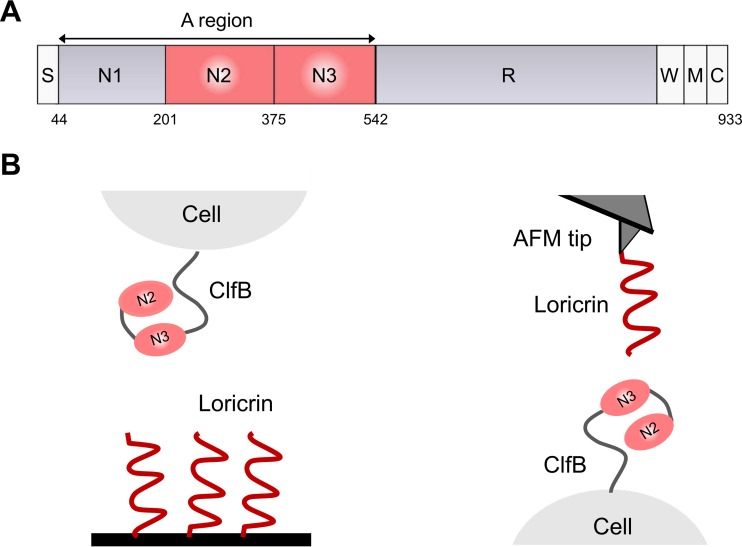
Force spectroscopy of the ClfB-Lor interaction. (A) Schematic diagram of the domain organization of ClfB showing the ligand-binding A region made of the N1, N2, and N3 subdomains; the serine-aspartate repeat region (R); the wall-spanning domain (W); the membrane anchor (M); and the cytoplasmic positively charged tail (C). (B) Analysis of the ClfB-Lor interaction by SCFS (left) and SMFS (right). For clarity, the N1 subdomain of ClfB is not shown (see the text for details).

*In vitro* biochemical analysis and animal model studies have revealed that the main target ligand for ClfB is the squamous epithelial cell envelope protein loricrin (here Lor) (10). This molecule is composed of Gly-Ser-rich regions, and the highest-affinity binding site for ClfB is located within Lor loop region 2 (10). ClfB was recently shown to be a major adhesin for the interaction of *S. aureus* with skin corneocytes from patients with the common inflammatory skin disease AD, suggesting that it may represent an interesting target for reducing skin colonization (11). ClfB contains an N-terminal ligand-binding A domain followed by a flexible stalk formed by repeats of the dipeptide serine-aspartate (Fig. 1A). The A domain is composed of three separately folded subdomains N1, N2, and N3. The N2 and N3 subdomains bind to cytokeratin 10, Lor, and fibrinogen (Fg) via the high-affinity “dock, lock, and latch” (DLL) multistep mechanism first described for the Fg-binding proteins SdrG and ClfA (12–15). Following the insertion of a short peptide sequence of Lor into a hydrophobic trench formed between the N2 and N3 subdomains of ClfB, a conformational change at the C terminus of N3 locks the peptide in place (12–15).

Despite the biological importance of the ClfB-Lor interaction, two important questions still remain, i.e., what the specific forces involved in DLL binding are and whether the adhesive properties of ClfB can be modulated by mechanical tension. Here, we addressed these issues by using single-cell and single-molecule atomic force microscopy (AFM) (16, 17). We measured the binding strength of full-length ClfB in laboratory strain Newman and in clinical isolate AD08, as well as that of recombinant ClfB N2 and N3 subdomains. To identify the binding sites within Lor that are important for the interaction, we compared the behavior of full-length Lor and that of L2v, a variant of Lor loop region 2 that supports ClfB-dependent adhesion. The results demonstrate that single ClfB-Lor bonds are remarkably strong (~1,500 pN), consistent with a high-affinity DLL mechanism. Full-length Lor and L2v interactions with ClfB show similar binding strengths, showing that L2v represents the main binding site. The ClfB-Lor interaction is strengthened by tensile loading, consistent with a model in which mechanical force activates a conformational switch in ClfB from a weakly binding folded state to a strongly binding extended state.

## RESULTS

### Binding forces between bacteria and loricrin.

**FIG 2:**
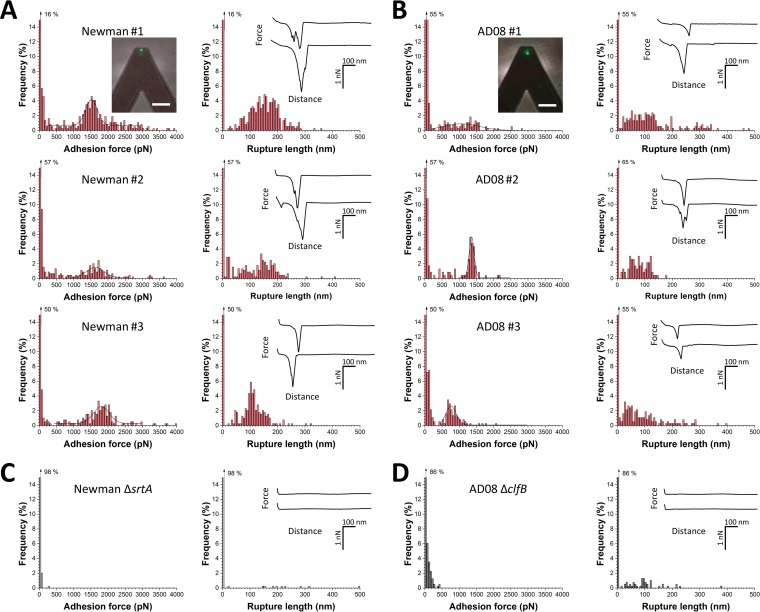
SCFS shows that ClfB mediates bacterial adhesion to Lor through weak and strong bonds. (A, B) Adhesion force and rupture length histograms with representative force curves obtained by recording force-distance curves in PBS between different Newman (A) or AD08 (B) cells and Lor substrates. Fluorescence images of the bacterial probes stained with the BacLight viability kit (insets in the upper panels; scale bars: 20 µm) demonstrate that the cell membrane is intact and thus that the assay is nondestructive. (C, D) Data obtained under the same conditions with cells of the Newman Δ*srtA* (C) and AD08 Δ*clfB* (D) strains. All curves were obtained by using a contact time of 100 ms, a maximum applied force of 250 pN, and an approach and retraction speed of 1,000 nm ⋅ s^−1^.

We initially investigated the forces involved in the adhesion of whole bacteria to Lor by using single-cell force spectroscopy (SCFS; Fig. 1B, left). *S. aureus* cells were immobilized on AFM cantilevers, and force-distance curves were recorded between the bacterial probes and Lor-coated substrates. In Fig. 2A, we present the adhesion forces and rupture distances recorded for three representative Newman cells (including cells from independent cultures; for more cells, see Table 1). Many force curves featured adhesion events with either weak forces (<500 pN) or strong forces (>1,000 pN). Most adhesive forces were missing from cells of the Δ*srtA* mutant Newman strain deficient in sortase A (Fig. 2C), indicating that CWA proteins are involved in the interaction. Using a macroscopic adhesion assay, Mulcahy et al. (10) found that, unlike wild-type cells, ClfB-deficient mutant Newman strain cells did not adhere to Lor. This strongly suggests that the forces in Newman cells originate from specific ClfB-Lor interactions.

**TABLE 1:** Probability of adhesion, mean maximum adhesion force, and mean rupture length measured in SCFS experiments with Lor substrates and cells of the *S. aureus* Newman wild-type and *ΔsrtA* mutant and AD08 wild-type and Δ*clfB* mutant strains^a^

Cell or parameter	Newman				AD08			
	*n*	*P*_adh_ (%)^b^	*F*_adh_ (pN)^c^	*L*_rupt_ (nm)^d^	*n*	*P*_adh_ (%)	*F*_adh_ (pN)	*L*_rupt_ (nm)
Wild type								
Cell 1	77	22	1,291 ± 970	102 ± 49	111	31	787 ± 630	58 ± 47
Cell 2	310	89	1,450 ± 863	155 ± 58	136	35	219 ± 274	87 ± 88
Cell 3	304	87	1,260 ± 1,015	162 ± 54	117	30	260 ± 249	372 ± 181
Cell 4	193	43	1,080 ± 845	126 ± 75	268	14	387 ± 440	91 ± 43
Cell 5	197	50	1,480 ± 801	116 ± 44	46	12	126 ± 132	36 ± 44
Cell 6	141	41	737 ± 392	136 ± 62	47	12	103 ± 77	68 ± 77
Cell 7	243	69	658 ± 321	139 ± 66	78	20	481 ± 358	97 ± 95
Cell 8					156	40	344 ± 274	299 ± 184
Cell 9					153	41	521 ± 415	98 ± 76
Cell 10					66	17	192 ± 162	138 ± 100
Cell 11					62	17	441 ± 361	113 ± 61
Cell 12					128	33	317 ± 423	116 ± 112
Mean		57 ± 25	1,136 ± 328	134 ± 21		25 ± 11	348 ± 192	131 ± 100
Mutant								
Cell 1	10	2	87 ± 60	224 ± 125	60	15	255 ± 152	257 ± 269
Cell 2	4	1	195 ± 141	9 ± 1	36	9	91 ± 55	71 ± 127
Cell 3	14	4	131 ± 230	65 ± 91	57	14	139 ± 87	313 ± 652
Cell 4	10	3	148 ± 83	46 ± 32	47	12	150 ± 123	115 ± 178
Cell 5					32	9	236 ± 130	83 ± 176
Cell 6					32	8	135 ± 125	70 ± 70
Mean		3 ± 1	140 ± 45	86 ± 95		11 ± 3	168 ± 64	151 ± 106

a *P*_adh_ and *F*_adh_ values of wild-type and mutant cells are statistically significantly different at the 0.05 level according to an unpaired *t* test. All values are the mean ± SD from *n* adhesive curves.

b *P*_adh_, percentage of curves with adhesion forces.

c *F*_adh_, maximum adhesion force.

d *L*_rupt_, rupture length.

**FIG 3:**
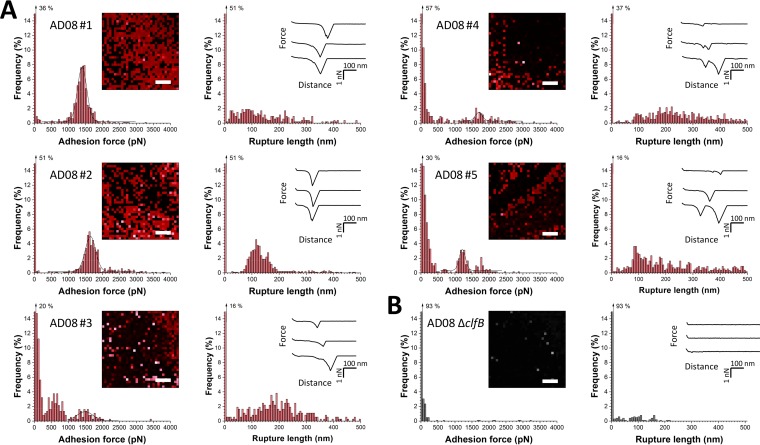
SMFS captures the localization and binding strength of ClfB on living bacteria. (A) Adhesion force and rupture length histograms obtained by recording force curves in PBS across the surface of five AD08 cells with tips labeled with Lor. The insets show adhesion force maps (left; scale bars, 100 nm; color scales, 4,000 pN) and representative force curves (right). Each red pixel represents the detection of a single adhesin (sometimes multiple adhesins). (B) Data obtained under the same conditions with an AD08 Δ*clfB* mutant cell. All curves were obtained by using a contact time of 100 ms, a maximum applied force of 250 pN, and an approach and retraction speed of 1,000 nm ⋅ s^−1^.

Strong forces slightly varied from one cell to another, but their distribution was always sharp and centered at 1,513 ± 208 pN (mean ± standard deviation [SD] of 254 adhesive curves), 1,634 ± 233 pN (95 curves), and 1,795 ± 252 pN (160 curves) for cells 1 to 3, respectively. These values are larger than those typically observed for bacterial adhesins, suggesting that the ClfB-Lor interaction is very strong. We believe that strong forces are due to the rupture of single bonds, rather than multiple weak bonds, because (i) different cells showed similar narrow distributions of strong forces; (ii) similar forces were detected by single-molecule force spectroscopy (SMFS) with Lor-coated AFM tips (Fig. 3, see below); (iii) earlier experiments have indicated that ClfB binds Lor via a high-affinity DLL mechanism (10, 14, 15); and (iv) in line with this, forces are in the range of values reported for single DLL interactions between SdrG and Fg (18).

The bacterium-Lor bonds ruptured at 136 ± 60 nm (mean ± SD of 692 adhesive curves based on three cells), which is shorter than the length of unfolded ClfB proteins. Lor molecules were immobilized on the substrates through multiple sites, meaning that they should not substantially contribute to the extensions measured. As the processed adhesin is made of 860 residues and its folded length is ~25 nm, protein unfolding should give an extension of ~285 nm. This shows that cell-Lor bonds rupture before complete unfolding of ClfB, suggesting that it is mechanically stable.

### Forces in a clinically relevant *S. aureus* strain.

We asked whether the Lor-binding forces measured in the laboratory strain also apply to the adhesion of a clinically relevant strain. We therefore analyzed the AD08 strain isolated from patients with the inflammatory skin disease AD (19). We recently demonstrated that ClfB is present in the cell wall of clinical isolate AD08 by performing Western blotting with anti-ClfB IgG (11). To show that the protein is active, adhesion to L2v was measured (11). A *clfB* mutant did not adhere to L2v, and adhesion was restored by complementation with a multicopy plasmid carrying *clfB*, indicating that ClfB is functional in AD08 (11). As shown in Fig. 2B, AD08 cells featured adhesive events with weak forces (<500 pN) and strong forces (>500 pN). This bimodal force distribution is qualitatively similar to that observed on Newman cells. However, analysis of multiple cells (Table 1) revealed differences between the two strains that may result from differences in growth conditions and in the surface density and conformation of the adhesins. Adhesion forces were strongly reduced on mutant cells lacking ClfB (AD08 Δ*clfB*) (Fig. 2D), indicating that they mostly originate from ClfB-Lor bonds. There was a small proportion of weak bonds on AD08 Δ*clfB* mutant cells that could reflect nonspecific interactions or binding to Lor by other CWA proteins. As ClfB promotes the adhesion of *S. aureus* to corneocytes from AD patients, ClfB-Lor interaction forces may play an important role in skin colonization and infection and could represent a potential target for therapy. Together, these observations show that ClfB is the key CWA protein involved in Lor binding by *S. aureus*.

### Binding strength and localization of single ClfB proteins.

Next, SMFS with Lor-modified tips was used to measure the strength of single ClfB-Lor bonds and to map their distribution on living bacteria (Fig. 1B, right). Shown in Fig. 3A are the adhesion force maps, adhesion forces, and rupture lengths obtained with Lor tips and five AD08 cells, including cells from independent cultures (for more cells, see Table 2). As in SCFS experiments, two types of adhesion profiles were observed, namely, weak forces (<500 pN) and strong forces (>1,000 pN; Gaussian distribution centered at 1,407 ± 137 pN [*n* = 608], 1,652 ± 307 pN [*n* = 474], 1,490 ± 238 pN [*n* = 59], 1,217 ± 184 pN [*n* = 157], and 1,699 ± 105 pN [*n* = 92] for cells 1 to 5). The magnitudes of the weak and strong forces and their relative proportions varied from one cell to another, presumably reflecting heterogeneity of the cell population. A major drop in adhesion probability was observed when the cells were blocked with free Lor (Fig. S1) or with AD08 Δ*clfB* mutant cells (Fig. 3B), showing that the measured adhesion forces involve specific ClfB-Lor bonds. Rupture lengths of up to 500 nm were observed, which is greater than the values from SCFS experiments (Fig. 2). This suggests that when individual adhesins are pulled by a strong force, they can be unraveled completely.

10.1128/mBio.01748-17.1 FIG S1  Blocking experiments demonstrate the specificity of the ClfB-Lor interaction, as measured by SMFS. (A) Adhesion force and rupture length histograms obtained by recording force curves in PBS across the surface of two AD08 cells with tips labeled with Lor. (B) Data obtained with the same cells 15 min after injection of free Lor at a final concentration of 10 µg ⋅ ml^−1^. All curves were obtained by using a contact time of 100 ms, a maximum applied force of 250 pN, and an approach and retraction speed of 1,000 nm ⋅ s^−1^. Download FIG S1, PDF file, 0.2 MB. Copyright © 2017 Vitry et al. 2017 Vitry et al. This content is distributed under the terms of the Creative Commons Attribution 4.0 International license.

**TABLE 2:** Probability of adhesion, mean maximum adhesion force, and mean rupture length measured in SMFS experiments with Lor tips and cells of the *S. aureus* AD08 wild-type and Δ*clfB* mutant strains^a^

Cell or parameter	*n*	*P*_adh_ (%)^b^	*F*_adh_ (pN)^c^	*L*_rupt_ (nm)^d^
Wild type				
Cell 1	481	47	1,700 ± 726	210 ± 116
Cell 2	665	65	1,360 ± 360	392 ± 1,159
Cell 3	1,006	50	1,710 ± 534	151 ± 83
Cell 4	860	84	556 ± 562	204 ± 110
Cell 5	501	49	803 ± 904	150 ± 108
Cell 6	716	70	564 ± 637	496 ± 1,700
Cell 7	440	43	803 ± 904	150 ± 108
Cell 8	849	83	364 ± 496	254 ± 144
Cell 9	860	84	917 ± 824	220 ± 134
Cell 10	901	88	1,070 ± 766	261 ± 107
Mean		66 ± 18	985 ± 472	113 ± 36
Mutant				
Cell 1	276	27	76 ± 60	215 ± 198
Cell 2	307	30	206 ± 231	116 ± 75
Cell 3	71	7	413 ± 926	95 ± 51
Cell 4	480	47	873 ± 580	136 ± 72
Cell 5	163	16	201 ± 375	127 ± 120
Mean		25 ± 15	354 ± 314	138 ± 46

a *P*_adh_ and *F*_adh_ values of wild-type and mutant cells are statistically significantly different at the 0.05 level according to an unpaired *t* test.

b *P*_adh_, percentage of curves with adhesion forces.

c *F*_adh_, maximum adhesion force.

d *L*_rupt_, rupture length.

Several observations support the notion that the ~1,500-pN forces represent the strength of single bonds. First, single rupture peaks with a rather narrow force distribution were observed. When multiple bonds rupture in parallel, a wider force range corresponding to multiples of the weakest unit forces is expected. Second, dilution of the density of Lor molecules attached to the tip (from 10 to 1%) dramatically decreased the adhesion frequency (Fig. S2) but without showing much intermediate forces. In the case of multiple bonds, dilution of the ligand is expected to lead to weaker forces corresponding to the unit force of single bonds. Third, strong forces are in the range of force values measured for single high-affinity DLL interactions between SdrG and Fg (18). In summary, our single-molecule experiments demonstrate that the ClfB-Lor complex is extremely stable, with a strength equivalent to that of a covalent bond (20). Given the strong forces we measured, one may argue that the polypeptide backbones of the receptor or ligand should break upon pulling. However, this should deactivate the AFM tip after a few force curves, which we never observed, indicating that receptor-ligand bonds were indeed measured. Strong forces agree with earlier data showing that ClfB binds Lor via a high-affinity DLL mechanism (10) and explain the ability of staphylococci to colonize the skin and the nose. We postulate that weak and strong forces are associated with two distinct binding states of ClfB engaged in the DLL mechanism. Force maps captured the localization of individual ClfB proteins on the bacteria, revealing that they were exposed at rather high density (Fig. 3A). The detection frequency varied from ~40 to 80%, which may reflect differences in protein expression but also in their conformation and orientation. Proteins generally formed a heterogeneous distribution reminiscent of that of SdrG and FnBPA, which may strengthen Lor interactions through multivalent interactions.

10.1128/mBio.01748-17.2 FIG S2  Decreasing the Lor density on the AFM tip shows that single molecules are probed. Adhesion force and rupture length histograms were obtained by recording force curves in PBS across the surface of two AD08 cells with Lor tips functionalized at very low density (16-mercaptododecahexanoic acid at 1% instead of 10%). The adhesion frequency is dramatically decreased without showing intermediate forces. Download FIG S2, PDF file, 0.1 MB. Copyright © 2017 Vitry et al. 2017 Vitry et al. This content is distributed under the terms of the Creative Commons Attribution 4.0 International license.

### Recombinant N2 and N3 subdomains weakly bind to loricrin.

**FIG 4:**
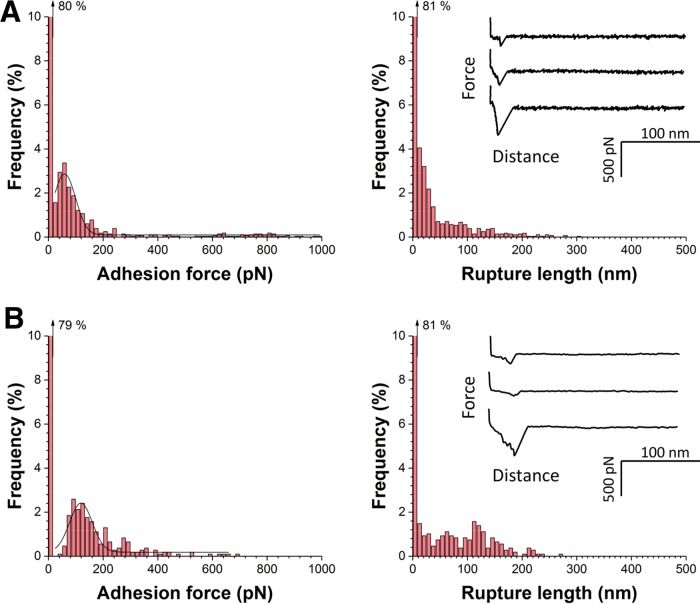
Recombinant ClfB_N2N3_ subdomains weakly bind to Lor. (A) Adhesion force and rupture length histograms with representative force curves obtained by recording force-distance curves in PBS between AFM tips bearing recombinant fragments corresponding to the ClfB_N2N3_ subdomains and Lor substrates. Fragments were immobilized on the tips by using NHS surface chemistry. (B) Data obtained under the same conditions by attaching recombinant fragments to the tips with a PEG-benzaldehyde linker. Data in panels A and B were pooled from three independents experiments. All curves were obtained by using a contact time of 150 ms, a maximum applied force of 250 pN, and an approach and retraction speed of 1,000 nm ⋅ s^−1^.

We also tested whether purified fragments of the ClfB ligand-binding region exhibit similar binding forces. Using SMFS, we measured the forces between Lor substrates and AFM tips functionalized with recombinant fragments corresponding to the N2 and N3 subdomains (amino acid residues 197 to 542) of ClfB. In Fig. 4A, we show data obtained when the fragments were attached via *N*-hydroxysuccinimide (NHS) surface chemistry (pool of three independent tips and substrates). Surprisingly, only weak forces were observed, with a mean adhesion force of 192 ± 280 pN and a rupture length of 54 ± 56 nm (*n* = 576 force curves). Similar results were obtained when the fragments were immobilized with a linker known to favor single-molecule detection (21) (Fig. 4B). So, unlike full-length ClfB on living bacteria, immobilized N2 and N3 subdomains cannot engage in strong bonds. Presumably, when the ligand-binding domain is anchored to a surface without being properly oriented and exposed via the flexible stalk, conformational changes needed for the DLL mechanism are hindered. This points to an important biological function for the stalk region, that is, optimal exposure of the ligand-binding region on the outermost cell surface.

### The ClfB-loricrin bond is strengthened by mechanical force.

**FIG 5:**
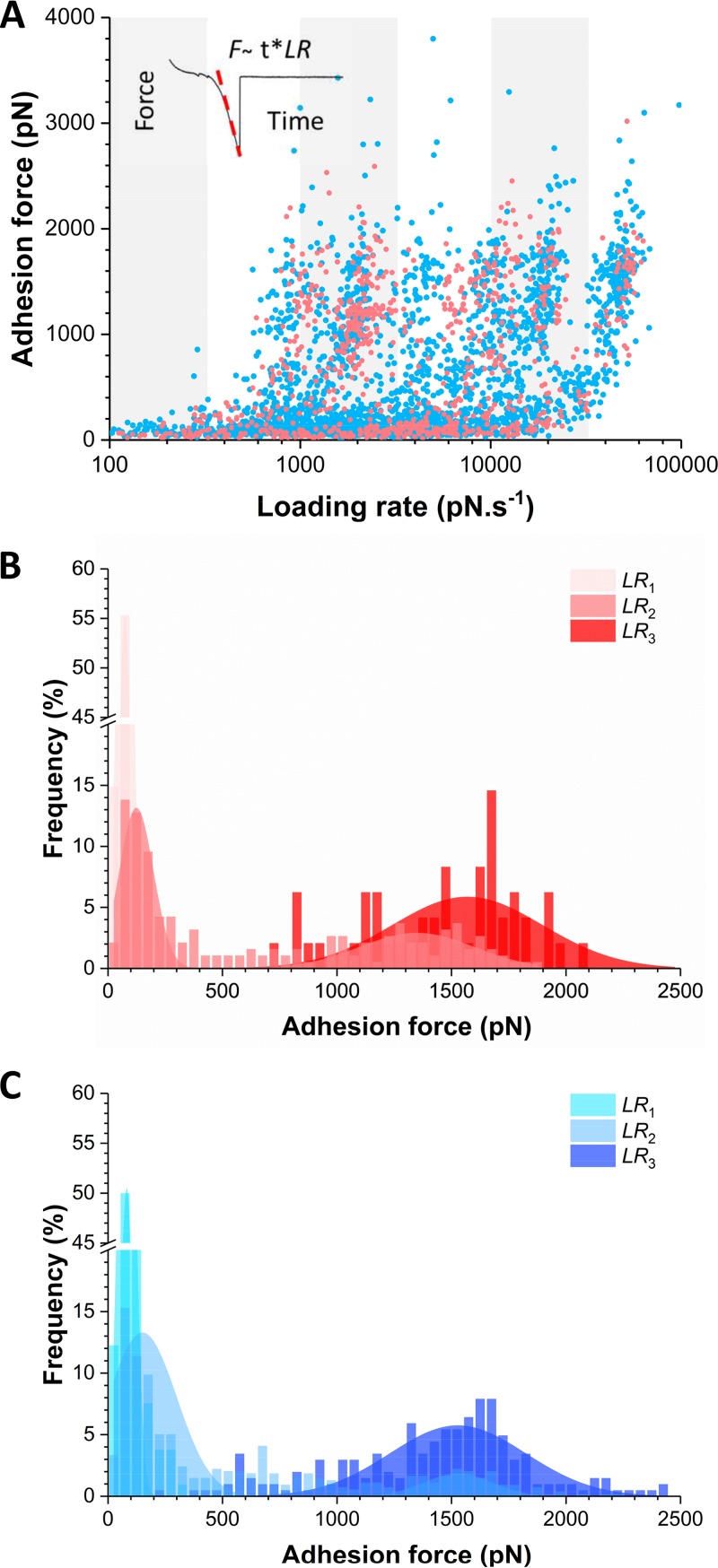
Mechanical force activates the ClfB-Lor interaction. (A) Dynamic force spectroscopy data showing the adhesion forces for ClfB-Lor (pink) and ClfB-L2v (blue) interactions measured at increasing LRs on AD08 cells (data pooled from 862 and 2,523 adhesive peaks on five cells for Lor and L2v, respectively). (B, C) To further analyze the results, small ranges of LRs were binned and the force distributions were plotted as histograms (see Fig. S3). This analysis reveals that for both Lor (B) and L2v (C), the dual-force distribution is switched, with the probability of forming strong bonds increasing with the LR (LR_1_ < 350 pN ⋅ s^−1^; 3,500 < LR_2_ <10,000 pN ⋅ s^−1^; LR_3_ > 35,000 pN ⋅ s^−1^).

We then analyzed the dynamics of the ClfB-Lor interaction on living bacteria by measuring the adhesion forces (F) while varying the loading rate (LR), i.e., the rate at which force is applied (Fig. 5). The effective LR was estimated from the force-versus-time curves to account for the contribution of cellular and protein elasticity (22). Figure 5A shows the resulting dynamic force spectroscopy plots for the Lor and L2v interactions. Adhesion forces showed cloudy distributions that were similar for both ligands, suggesting that they bind ClfB in a similar fashion. We further dissected the ligand-binding forces by analyzing their distribution over discrete ranges of LRs (Fig. S3). For ClfB-Lor bonds (Fig. 5B), the lowest LR showed only weak forces centered at 74 ± 60 pN, while the highest LR featured strong forces of 1,569 ± 656 pN without any evidence of weak forces. At intermediate LRs, weak and strong forces coexisted in various proportions, depending on the LR, while intermediate forces (in the range of 500 to 1,000 pN) were not frequently observed. The same behavior was observed for the ClfB-L2v bonds (Fig. 5C), again supporting the idea that this is the primary binding region. Accordingly, our data show that the probability of forming strong bonds increases with the LR, thus that the ClfB-mediated interaction strengthens with the applied force.

10.1128/mBio.01748-17.3 FIG S3  The probability of forming strong bonds increases with the LR. (A, B) Adhesion forces between AD08 cells and Lor or L2v tips were measured at various LRs. Small ranges of LRs were binned, and the distribution of the Lor (A) and L2v (B) forces was plotted as histograms (data pooled from five independent experiments for both Lor and L2v). Download FIG S3, PDF file, 0.2 MB. Copyright © 2017 Vitry et al. 2017 Vitry et al. This content is distributed under the terms of the Creative Commons Attribution 4.0 International license.

**FIG 6:**
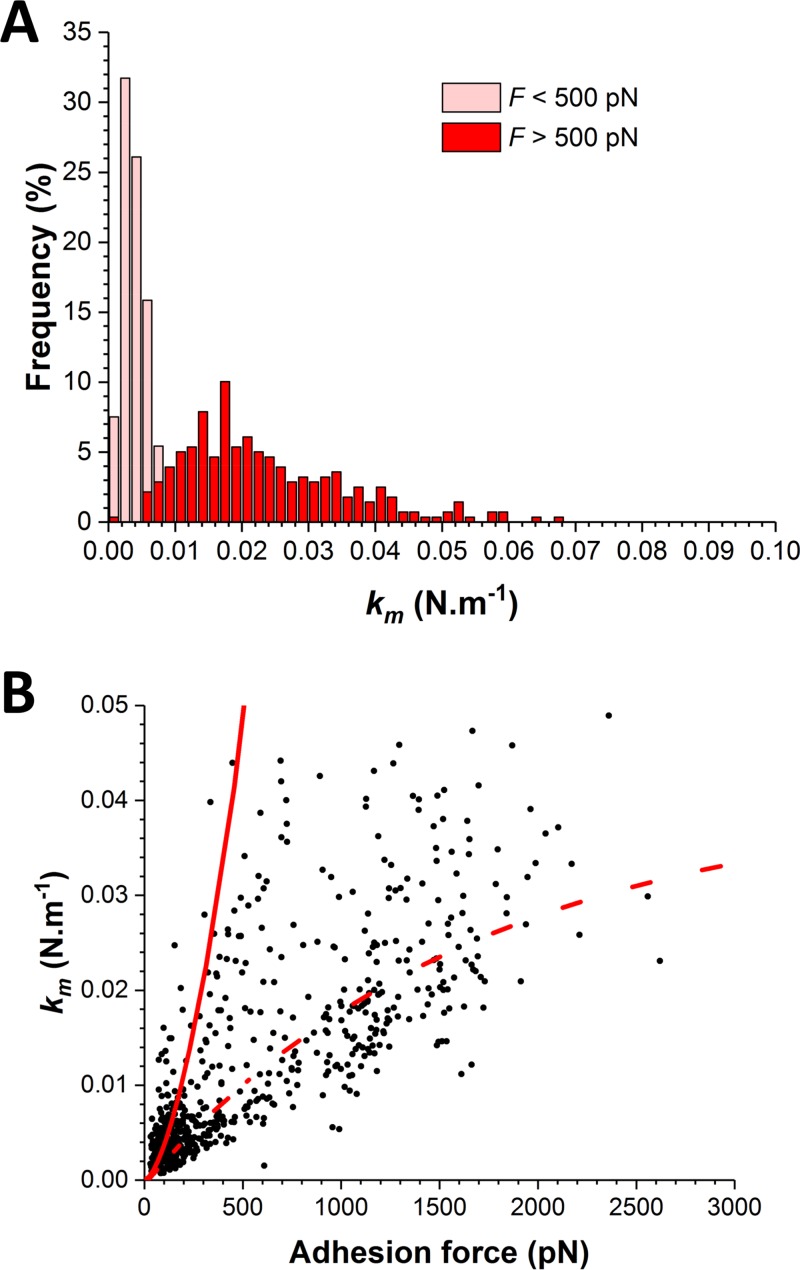
The switch in force distribution correlates with an increase in molecular stiffness. (A) Distribution of the spring constants of the molecular complex (*k*_*m*_) at low (<500 pN) and high (>500 pN) force levels. (B) Plot of *k*_*m*_ versus adhesion force values together with simulations based on a WLC model taking into account the elasticity of the ClfB protein only (continuous line) or that of the protein and the cell wall (dashed line).

What is the molecular origin of this switch in force distribution? As the strengths of weak and strong bonds largely differ, there is no obvious reason why strong bonds would be due to the simultaneous rupture of multiple weak bonds. In such a case, intermediate forces resulting from double or triple bonds should be much more frequently observed and lead to more complex distributions. A more likely explanation is that the shift toward strong forces results from a change in the conformational state of ClfB from a weakly to a strongly binding state, as observed for catch bonds (23). Supporting this view, we found that the switch in force distribution was correlated with an increase in molecular stiffness (*k*_*m*_) (Fig. 6). We estimated the spring constant of the molecular complex (*k*_*m*_) by using the slope (*s*) of the linear portion of the raw deflection-versus-piezo displacement curves and the equation *k*_*m*_ = (*k*_c_ × *s*)/(1 − *s*), where *k*_c_ is the spring constant of the AFM cantilever. For weak and strong forces, we found that *k*_*m*_ = 3.5 ± 0.1 pN nm^−1^ and *k*_*m*_ = 18 ± 1 pN nm^−1^, respectively, indicating that strong forces were associated with higher molecular stiffness. Modeling of the F-versus-*k*_*m*_ plot by using a worm-like chain (WLC) model revealed that *k*_*m*_ values at a low force level are consistent with the molecular elasticity of the ClfB protein, whereas those at a high force level may reflect the elasticity of two springs, the protein and the cell wall, in series (22). This suggests that, unlike weak forces, strong forces propagate through the entire protein to be transmitted to the cell wall. Together, these observations support the notion that the application of an external force triggers a conformational change in ClfB from a folded, weakly binding state to an extended, strongly binding state. We anticipate that the ability of AFM to subject single molecules to controlled force in living bacteria will help researchers to identify such force-dependent activation in other adhesins.

## DISCUSSION

Recently, it has become clear that bacterial behavior can be influenced by mechanical forces (24). Bacteria that attach to surfaces are subjected to various physical stresses, such as hydrodynamic flow and cell surface contacts. Mechanics plays important roles during the colonization of human tissues by pathogens like *S. aureus*. An important yet unsolved question is whether *S. aureus* adhesion can be modulated in response to mechanical stress. We have shown that the ClfB-Lor interaction is strengthened by tensile force. We propose that this force-sensitive mechanism, not yet described for any staphylococcal adhesin, provides the cells with a means to modulate their adhesive properties during the colonization of host surfaces. Our experiments emphasize the role of mechanics in driving the biological functions of *S. aureus* surface proteins.

The ClfB-Lor interaction features a bimodal force distribution, that is, weak bonds of ~250 pN and strong bonds of ~1,500 pN. Strong bonds are consistent with a high-affinity DLL binding mechanism involving dynamic conformational changes. L2v and full-length Lor have the same weak and strong binding forces, indicating that loop region 2 is the primary binding site for ClfB, with the other ligand regions playing a minor role. Recombinant ClfB N2 and N3 subdomains only show weak forces, presumably because immobilization of the ligand-binding domain on a surface does not allow sufficient freedom to undergo the conformational changes needed for the DLL. This points to a role for the flexible stalk region in projecting the binding sites away from the bacterial surface and enabling strong DLL binding.

The strong dissociation forces are among the strongest receptor-ligand bonds measured so far, which is in contrast to the rather classical biochemical affinity values (µM range) (10). This disagreement suggests that the unbinding pathway of ClfB under mechanical force strongly differs from that at equilibrium, i.e., in the absence of force, which was also recently reported for the ligand-receptor complex responsible for substrate anchoring in the *Ruminococcus flavefaciens* cellulosome (25). So the binding strength of adhesion proteins measured under tensile force at nonequilibrium may be completely uncorrelated with the bulk equilibrium affinity measured by classical bioassays. As adhering bacteria are often subjected to mechanical stresses, this suggests that binding forces are more relevant than affinities to describe bacterial adhesion under physiological conditions.

An important outcome of this study is that the ClfB-Lor bond strengthens with the tensile force. The force distribution switches with the rate at which force is applied to the bond; while weak bonds dominate at low stress levels, strong bonds are favored at high stress levels. The transition from weak to strong binding correlates with an increase in molecular stiffness. These observations are consistent with a two-state model in which ClfB-mediated adhesion is enhanced through force-induced conformational changes in the adhesin from a weakly binding folded state to a strongly binding extended state. The force-enhanced adhesion unraveled here is reminiscent of catch bonds, which enable weak adhesion at a low flow rate but strong adhesion at a high flow rate (23). In bacteria, the archetypal catch bond protein is the mannose-binding adhesin FimH of *Escherichia coli* (26). At a low force level, the FimH-mannose bond is weak and relatively short lived, while the bond is strengthened at a high force level. Several models have been proposed to explain catch bond mechanisms (23). In the two-pathway model, the ligand can escape from the receptor binding site through two alternative pathways, with either a low- or a high-energy barrier. The ligand will exit the high-energy barrier route only when sufficiently strong mechanical force is applied. In the allosteric model, force triggers a conformational change in the ligand-binding site, from a low- to a high-affinity state (26, 27). An example of such an allosterically regulated protein is FimH, where tensile mechanical force induces an allosteric switch to the high-affinity, strong binding conformation of the adhesin. Besides catch bonds, there seem to be alternative mechanisms of shear-enhanced adhesion that are less specific. For instance, adhesion of *Pseudomonas aeruginosa* to abiotic surfaces was shown to be enhanced by shear stress, an effect that involved multiple adhesive structures (pili, flagella, and polysaccharides) (28).

While our data favor a model in which force-enhanced adhesion involves conformational changes in the ClfB protein, the underlying molecular details are not clear. We postulate that the weak and strong dissociation forces may result from two competing unbinding pathways with different mechanical characteristics (29). For the mechanically stable multidomain cellulosome protein complex (29), steered molecular dynamics and single-molecule experiments revealed that strong forces can be achieved if the complex directs force along pathways nonparallel to the pulling direction. This model would explain why the ClfB-ligand complex is capable of withstanding forces equivalent to the mechanical strength of a covalent bond. That force is transmitted along stiff paths through the complex is supported by our molecular spring constants, revealing that under high loading, force is transmitted up to the peptidoglycan. It is tempting to speculate that ClfB could function as a mechanosensor capable of feeling mechanical forces acting on the cell wall and of triggering specific intracellular responses.

Our finding that mechanical force potentiates ClfB-mediated *S. aureus* adhesion is of biological relevance, as this represents a powerful means to modulate the strength of interaction with host tissues, helping cells to attach firmly under high shear stress and to detach and spread under low stress. During the colonization of surfaces such as the most squamous epithelium of the human nares, bacteria are subject to mechanical stress associated with fluid flow, scraping, or epithelial turnover (30). To withstand shear while they bind to host skin and mucosal surfaces, staphylococci use a collection of CWA proteins with various binding mechanisms (30). Our results suggest that these proteins may have evolved a sophisticated mechanism to strengthen adhesion under stress. This model is supported by flow experiments showing that high shear forces can enhance *S. aureus* adhesion. ClfA-dependent adhesion to immobilized platelets increases with the shear rate (31). *S. aureus*-promoted platelet activation involves Fg forming a bridge between ClfA on the bacterial cell surface and the platelet integrin (32, 33). During endovascular infections, *S. aureus* overcomes shear forces of flowing blood by attaching to von Willebrand factor (VWF) (34, 35). Shear-resistant adhesion involves a secreted staphylococcal VWF-binding protein that simultaneously interacts with ClfA on the bacterial cell surface and with VWF on the vessel wall (36). These examples show that increased shear stress can promote the adhesion of *S. aureus* and that this force-sensitive adhesion involves CWA proteins. In contrast, we expect that weak ClfB bonds observed under low physical stress will help the bacteria to detach easily and colonize new sites.

In conclusion, this study provides the first direct demonstration that mechanical tension can enhance the adhesion of a staphylococcal protein, which could represent a general mechanism that allows CWA proteins to tune their adhesive function in response to stress. Our results suggest that the binding strength of adhesion proteins—measured under tensile force at nonequilibrium—are more relevant than bulk equilibrium affinity—measured by traditional bioassays—to describe bacterial adhesion under physiological conditions. The two-state binding mechanism of ClfB—and possibly other adhesins as well—represents an interesting new target for antiadhesion therapy; the design of inhibitors able to prevent the transition from the low- to the high-binding state might contribute to the efficient inhibition of staphylococcal adhesion under physiological shear stress conditions.

## MATERIALS AND METHODS

### Bacterial strains and growth conditions.

Wild-type *S. aureus* strain Newman and its mutant deficient in sortase A, the enzyme responsible for anchoring CWA proteins to peptidoglycan (Newman Δ*srtA*) were cultured in Trypticase soy broth (TSB) overnight at 37°C under agitation. Before experiments, cells were washed twice in prewarmed TSB and then 50 μl of this solution was inoculated into 10 ml of fresh prewarmed TSB. Cells were grown at 37°C under agitation until an optical density at 600 nm of 0.3 was reached. *S. aureus* strain AD08, a clinical isolate from an AD patient, and its isogenic ClfB-deficient mutant AD08 Δ*clfB* (11) were cultured in TSB overnight at 37°C under agitation. For AFM experiments, cells were harvested by centrifugation, washed twice in phosphate-buffered saline (PBS), and diluted 1:100 in PBS.

### Purified proteins and recombinant fragments.

Recombinant glutathione *S*-transferase-tagged Lor and L2v were purified from *E. coli* with a GSTrap FF purification column (GE Healthcare) in accordance with the manufacturer’s instructions as previously described (10). Recombinant ClfB N2N3 (residues 201 to 542, Newman sequence) were purified from *E. coli* by nickel affinity chromatography as previously described (10).

### Functionalization of substrates and cantilevers with loricrin.

Gold-coated glass coverslips and cantilevers (OMCL-TR4; Olympus, Tokyo, Japan; nominal spring constant, ~0.02 N ⋅ m^−1^) were immersed overnight in an ethanol solution containing 1 mM 10% 16-mercaptododecahexanoic acid--90% 1-mercapto-1-undecanol (Sigma), rinsed with ethanol, and dried with N_2_. Substrates and cantilevers were then immersed for 30 min in a solution containing 10 mg ⋅ ml^−1^ NHS and 25 mg ⋅ ml^−1^ 1-ethyl-3-(3-dimethylaminopropyl)-carbodiimide (Sigma), rinsed with ultrapure water (ELGA LabWater), incubated with 0.1 mg ⋅ ml^−1^ Lor for 1 h, rinsed further with PBS buffer, and then immediately used without dewetting. For some experiments, cantilevers were functionalized with L2v instead of Lor.

### Functionalization of cantilevers with ClfB_N2N3_.

For experiment with purified fragments, gold cantilevers (OMCL-TR4; Olympus, Tokyo, Japan) were functionalized with ClfB_N2N3_ via the NHS chemistry described above. In addition, oxide-sharpened microfabricated Si_3_Ni_4_ cantilevers (MSCT; Bruker) were also functionalized with ClfB_N2N3_ with polyethylene glycol (PEG) linkers as described elsewhere (21, 37).

### SCFS.

Bacterial cell probes were obtained as previously described (38, 39). Briefly, colloidal probes were obtained by attaching a single silica microsphere (6.1-μm diameter; Bangs Laboratories) with a thin layer of UV-curable glue (NOA 63; Norland Edmund Optics) to triangle-shaped tipless cantilevers (NP-O10; Bruker) with a NanoWizard III atomic force microscope (JPK Instruments, Berlin, Germany). The cantilevers were then immersed for 1 h in Tris-buffered saline (TBS; 50 mM Tris, 150 mM NaCl, pH 8.5) containing 4 mg ⋅ ml^−1^ dopamine hydrochloride (Sigma-Aldrich), rinsed in TBS, and used directly for cell probe preparation. The nominal spring constant of the colloidal probe was determined by the thermal noise method. A 50-μl volume of a diluted cell suspension was then deposited into a petri dish containing Lor-coated substrates at a distinct location within the petri dish, and 3 ml of PBS was added to the system. The colloidal probe was put in contact with a single bacterial cell and retracted to attach it to the silica microsphere; proper attachment of the cell to the colloidal probe was checked by optical microscopy. To check the viability and positioning of the cell, bacteria were stained with a BacLight viability kit (Invitrogen kit L7012) in accordance with the manufacturer’s instructions. Three microliters of a 1:1 Syto 9-propidium iodide mixture at 1.5 mM was added to 1 ml of the cell suspension, mixed thoroughly, and incubated for 15 min in the dark. Optical images were recorded on a Zeiss Axio Observer Z1 equipped with a Hamamatsu C10600 camera. Cell probes were used to measure interaction forces on Lor surfaces at room temperature with an applied force of 0.25 nN, a constant approach-retraction speed of 1.0 μm ⋅ s^−1^, and a contact time of 100 ms. Data were analyzed with the data processing software from JPK Instruments (Berlin, Germany). Adhesion force and rupture distance histograms were obtained by calculating the maximum adhesion force and the rupture distance of the last peak for each curve.

### SMFS.

SMFS measurements were performed at room temperature in PBS buffer with a NanoWizard III atomic force microscope (JPK Instruments, Germany). For cell experiments, bacteria were immobilized on polystyrene substrates. Adhesion maps were obtained by recording 32 × 32 force-distance curves on areas of 500 by 500 nm^2^ with an applied force of 250 pN, a constant approach and retraction speed of 1 µm ⋅ s^−1^, and a contact time of 100 ms. Experiments were also carried out while varying the retraction speed from 100 to 5 µm ⋅ s^−1^. On model surfaces, multiple force-distance curves were recorded in areas of 10 by 10 µm^2^ with an applied force of 250 pN, a constant approach and retraction speed of 1 µm ⋅ s^−1^, and a contact time of 150 ms. Adhesion force and rupture distance histograms were obtained by calculating the force and rupture distance of the last peak for each curve. The spring constants of the cantilevers were measured by the thermal noise method. Data were analyzed with the data processing software from JPK Instruments (Berlin, Germany).
