# Supplementary material for: Force-Induced Strengthening of the Interaction between Staphylococcus aureus Clumping Factor B and Loricrin
Source: mBio. 2017 Dec 5;8(6):e01748-17. doi: 10.1128/mBio.01748-17 (PMC5717387; doi:10.1128/mBio.01748-17)
Supplement: FIG S1 [file mbo006173618sf1.pdf]

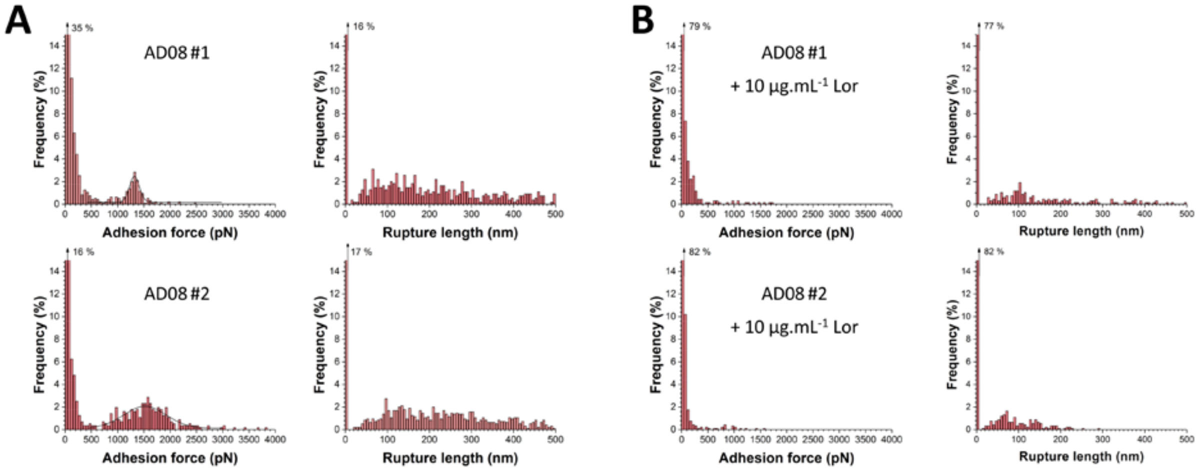

Fig. S1. Blocking experiments demonstrate the specificity of the ClfB-Lor interaction, as measured by single-molecule force spectroscopy. (*A*) Adhesion force and rupture length histograms obtained by recording force curves in PBS across the surface of two AD08 cells using tips labelled with Lor. (*B*) Data obtained on the same cells 15 min after injection of free Lor at a final concentration of 10 µg.mL^{-1}. All curves were obtained using a contact time of 100 ms, a maximum applied force of 250 pN, and approach and retraction speeds of 1,000 nm.s^{-1}. th Lor and L2v).
