# Supplementary material for: Force-Induced Strengthening of the Interaction between Staphylococcus aureus Clumping Factor B and Loricrin
Source: mBio. 2017 Dec 5;8(6):e01748-17. doi: 10.1128/mBio.01748-17 (PMC5717387; doi:10.1128/mBio.01748-17)
Supplement: FIG S2 [file mbo006173618sf2.pdf]

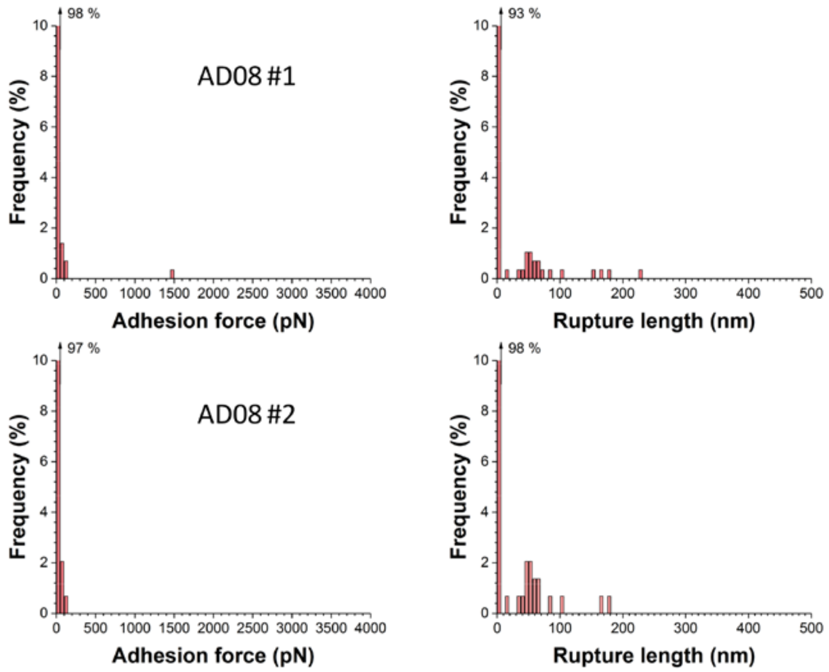

Fig. S2. Decreasing the Lor density on the AFM tip shows that single-molecules are probed. Adhesion force and rupture length histograms obtained by recording force curves in PBS across the surface of two AD08 cells using Lor-tips functionalized at very low density (16-mercaptododecahexanoic acid at 1% instead of 10%). The adhesion frequency is dramatically decreased, without showing intermediate forces.
