# Supplementary material for: Force-Induced Strengthening of the Interaction between Staphylococcus aureus Clumping Factor B and Loricrin
Source: mBio. 2017 Dec 5;8(6):e01748-17. doi: 10.1128/mBio.01748-17 (PMC5717387; doi:10.1128/mBio.01748-17)
Supplement: FIG S3 [file mbo006173618sf3.pdf]

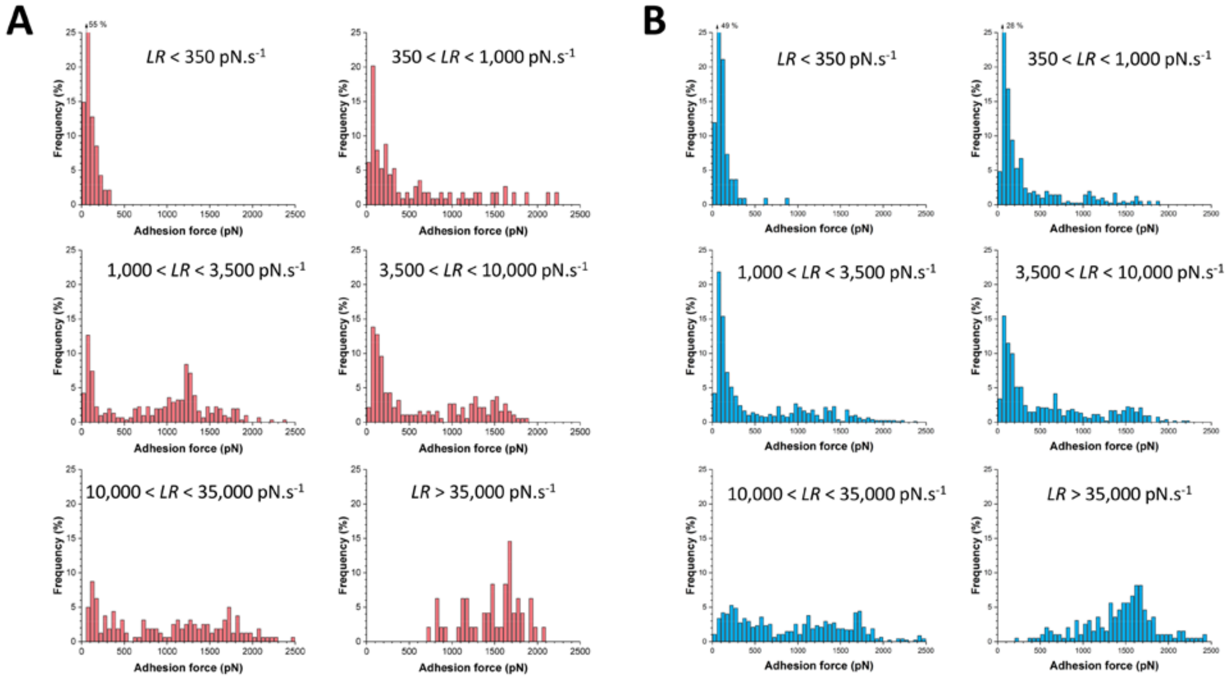

Fig. S3. The probability of forming strong bonds increases with the loading rate. (*A*, *B*) Adhesion forces were measured at various loading rates (*LRs*) between AD08 cells and Lor- or L2v-tips. Small ranges of *LRs* were binned and the distribution of the Lor (*A*) and L2v (*B*) forces plotted as histograms (data pooled from 5 independent experiments for both Lor and L2v).
